# Development of a Real-Time Microchip PCR System for Portable Plant Disease Diagnosis

**DOI:** 10.1371/journal.pone.0082704

**Published:** 2013-12-12

**Authors:** Chiwan Koo, Martha Malapi-Wight, Hyun Soo Kim, Osman S. Cifci, Vanessa L. Vaughn-Diaz, Bo Ma, Sungman Kim, Haron Abdel-Raziq, Kevin Ong, Young-Ki Jo, Dennis C. Gross, Won-Bo Shim, Arum Han

**Affiliations:** 1 Department of Biomedical Engineering, Texas A&M University, College Station, Texas, United States of America; 2 Department of Plant Pathology and Microbiology, Texas A&M University, College Station, Texas, United States of America; 3 Department of Electrical and Computer Engineering, Texas A&M University, College Station, Texas, United States of America; New York University, United States of America

## Abstract

Rapid and accurate detection of plant pathogens in the field is crucial to prevent the proliferation of infected crops. Polymerase chain reaction (PCR) process is the most reliable and accepted method for plant pathogen diagnosis, however current conventional PCR machines are not portable and require additional post-processing steps to detect the amplified DNA (amplicon) of pathogens. Real-time PCR can directly quantify the amplicon during the DNA amplification without the need for post processing, thus more suitable for field operations, however still takes time and require large instruments that are costly and not portable. Microchip PCR systems have emerged in the past decade to miniaturize conventional PCR systems and to reduce operation time and cost. Real-time microchip PCR systems have also emerged, but unfortunately all reported portable real-time microchip PCR systems require various auxiliary instruments. Here we present a stand-alone real-time microchip PCR system composed of a PCR reaction chamber microchip with integrated thin-film heater, a compact fluorescence detector to detect amplified DNA, a microcontroller to control the entire thermocycling operation with data acquisition capability, and a battery. The entire system is 25×16×8 cm^3^ in size and 843 g in weight. The disposable microchip requires only 8-µl sample volume and a single PCR run consumes 110 mAh of power. A DNA extraction protocol, notably without the use of liquid nitrogen, chemicals, and other large lab equipment, was developed for field operations. The developed real-time microchip PCR system and the DNA extraction protocol were used to successfully detect six different fungal and bacterial plant pathogens with 100% success rate to a detection limit of 5 ng/8 µl sample.

## Introduction

Crop production worldwide is highly vulnerable to emerging and variable pathogens that can cause devastating economic losses and threaten global food security [1]. The introduction of exotic plant pathogenic microbes has continually challenged US agriculture, *e.g*., recent threats to citrus production in Florida, soybean production in the Midwest, and native stands of oaks and forests in California [2]. The ornamental industry in Texas is vulnerable to importation of quarantined pathogens as a result of the large-scale movement of soil and plant material from out-of-state as well as offshore producers [3]. Federal and state regulators recently responded to the introduction of *Phytophthora ramorum* and *Ralstonia solanacearum* race 3 biovar 2 (R3B2) on ornamentals to quarantine and eradicate infected plants. To meet these new and growing challenges caused by plant pathogenic microbes, there is a critical need for the development of innovative technologies for rapid, accurate, and cost-effective detection and identification of such etiologic agents in field settings.

A variety of commercial kits and methods are currently available for microbial pathogen detection and diagnosis such as ELISA kit (Invitrogen, Grand Island, NY, USA) and ViaLight™ kit (Lonza, Basel, Switzerland). The majority of these test kits on the market is antigen/antibody-based immunoassays and lack high-throughput capability or high sensitivity. Nucleic acid-based pathogen detection technologies can overcome these limitations with the use of DNA amplification for species-specific and pathovar-specific detection of microbes [4]. These DNA-based detection systems include polymerase chain reaction (PCR), strand-displacement amplification (SDA), nucleic acid sequence-based amplification (NASBA), rolling-circle amplification (RCA) and the Q-beta replicase reaction [5]. Notably, PCR has been widely accepted and used for pathogen detection and diagnostics due to its ease of use, accuracy and availability. However, conventional bench-top PCR systems are not suitable for portable operations not only due to their size and cost, but also due to the requirement of gel electrophoresis in a laboratory setting to quantify and detect the amplified DNA (amplicon). A more advanced form of PCR, *i.e*., real-time PCR, has emerged as the diagnostic tool of choice in recent years to rapidly identify and quantify critical pathogenic agents [6]–[9]. While conventional PCR and real-time PCR systems are both widely accepted and popular in plant disease diagnostics, due to their need for sample preparation, power supply, and logistics, they are not considered for on-field operation.

Recent advances in microfluidics and micro/nano fabrication technologies led to the development of various miniaturized PCR systems suitable for biological applications [5], [10]–[12]. These systems utilized unique characteristics of microtechnologies, such as small sample/reagent volume, large surface to volume ratio, and compact size for fast and accurate analysis of biological samples at low cost. These microchip PCR systems typically have very small reaction chambers that contain micro- to nano-liter scale reagents, significantly reducing the time for each thermal cycling step. However, these systems do not employ real-time detection, and thus require additional methods or devices such as gel electrophoresis for the quantification of amplicons. In recent years, several real-time microchip PCR systems integrated with optical detection systems have been developed with promising results, but mostly for clinical diagnosis applications [13]–[22], Julich *et al.* has reported the one real-time microchip PCR system for plant pathogen detection [23], but the system still requires various auxiliary instruments such as a laptop, fluidic pump, electrical readout and temperature regulation system which are larger than the laptop size.

Another roadblock in field deployable real-time PCR systems is the lack of suitable high quality DNA extraction protocols that can allow the users to circumvent the requirement for laboratory instruments. Typically, diseased plant samples are brought into a diagnostic lab where liquid nitrogen is used to grind and extract DNA from the samples [24]–[26]. One of the key needs for a truly portable plant pathogen diagnosis microchip PCR system is to develop a method that would allow users to prepare PCR-grade DNA samples without the standard in-lab practices that requires many different chemicals and liquid nitrogen.

Together, these limitations prevent microchip PCR systems from becoming a practical portable system of choice that can be deployed for on-field operation in plant pathogen diagnosis. Here, we present a portable real-time microchip PCR system to meet the future demand for on-field plant pathogen detection. The uniqueness of our system is the development of a highly compact fluorescence detector and a battery-powered microcontroller unit for PCR, rendering our instrument compact and portable. The entire system is 25×16×8 cm^3^ in size and weighs under 850 g, substantially smaller compared to other real-time microchip PCR systems that typically require a computer and several auxiliary instruments. In addition, a DNA sample preparation method was developed to allow users to rapidly and conveniently prepare DNA samples for real-time PCR in the field. The system was successfully demonstrated by detecting six different plant pathogens with 100% efficiency with a detection limit of 5 ng/8 µl sample. This real-time microchip PCR system and field deployable DNA extraction protocol will provide momentous inroads to the development of a truly portable, rapid, and practical microchip PCR system for on-field plant pathogen detection and diagnostics.

## Materials and Methods

### Real-time microchip PCR System Design & Fabrication

**Figure 1:**
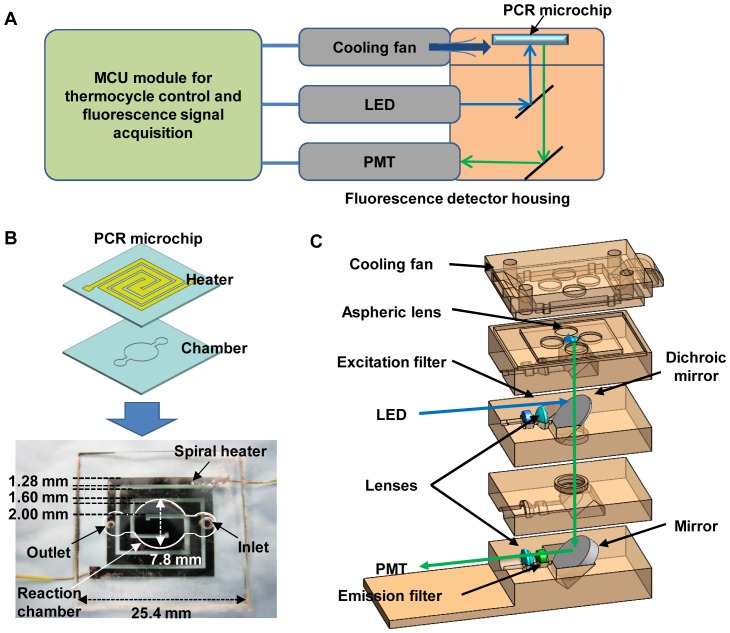
Illustration of the portable real-time microchip PCR system. (A) An overview of the real-time microchip PCR system, including a PCR microchip, a fluorescence detector housing coupled to an LED and a PMT, a cooling fan to accelerate cooling, and a microcontroller unit (MCU) for thermocycle control and fluorescence signal acquisition. (B) The PCR microchip is composed of a heater layer and a reaction chamber layer bonded together. The widest line of the heater is located at the center of the chamber, and the widths gets bigger by 1.25 times from the outermost trace to the center trace, thus are 1280, 1600, 2000, and 2500 µm. The gap between the spiral heater lines is 630 µm. The diameter and depth of the circular reaction chamber is 7.8 mm and 80 µm, respectively. White dotted line in the image shows the location of the reaction chamber and the inlet/outlet. (C) Schematic of the compact fluorescence detector housing and optical components inside the housing.

The real-time microchip PCR system is composed of three functional units: a PCR microchip for amplifying PCR samples, an optical setup for detecting amplified DNA in real time, and a control/data acquisition part for monitoring and controlling the entire real-time PCR process (Figure 1A).

PCR microchip. The PCR microchip was made up of two 25.4×25.4×0.5 mm^3^ glass slides, where the bottom slide has a stationary reaction chamber holding the PCR sample and the top slide has a thin film heater to precisely and uniformly control the reaction chamber temperature (Figure 1B). The circular reaction chamber in the bottom glass slide (diameter: 7.8 mm, depth: 80 µm) holds 8-µl of PCR sample to be amplified. Two different size holes were made as the inlet and outlet (2 mm and 1 mm in diameter, respectively) of the reaction chamber, in order to load samples into the reaction chamber through the 2 mm hole and to extract amplified samples from the reaction chamber through the 1 mm hole for off-chip validation. When loading samples through a 2 mm hole, samples can easily fill the entire reaction chamber due to capillary force. However when injecting samples through a 1 mm hole that tightly fits to a pipette tip (1–200 µl, VWR, Sugar Land, TX, USA), samples tend to flow only along one side of the reaction chamber from time to time and did not fill the entire reaction chamber so that an air bubble is formed in the chamber. This air bubble expands during thermocycling, making it difficult to properly measure the fluorescence of liquid sample. When extracting samples, the 1–200 µl pipette tip was tightly plugged into the 1 mm hole and vacuum was applied to completely extract the sample in the reaction chamber. By forming a tight seal between the 1 mm hole and the pipette tip, amplified samples were efficiently extracted.

To obtain a uniform temperature distribution over the entire reaction chamber during thermocycling, a square spiral shaped thin-film electrode (heater) was designed, similar to the one reported by Kim *et al.* [27]. The width of the outermost trace was 1280 µm, and the widths of the traces were widened by a ratio of 1.25 in succeeding traces towards the middle of the chamber (trace width from outside to middle: 1280, 1600, 2000 and 2500 µm). The gap between neighboring traces in the square spiral heater design was 630 µm. Gold (Au) was selected as the thin-film heater material, with chromium (Cr) being used as the adhesion layer between gold and the glass slide. The temperature profile of the PCR microchip when heated by the on-chip thin-film heater was simulated by a commercial finite element method (FEM) software (COMSOL Multiphysics®, COMSOL Inc., Los Angeles, CA, USA). Time-series thermal simulations were conducted while applying various voltages between the two ends of the heater traces to optimize the heater design to achieve uniform temperature.

The microchip was fabricated using standard microfabrication technologies. Briefly, for the heater fabrication, Cr and Au layers (25 and 200 nm thick) were deposited on a clean borofloat glass (50.8×50.8×0.5mm^3^) using an e-beam evaporator. Photoresist (Microposit™ S1818, Rohm-Haas-Shipley Company, Inc., Marlborough, MA, USA) was spin-coated on it, and the square spiral heater was patterned on the photoresist layer using photolithography. Selective metal etch using Cr and Au etchants was followed by photoresist removal. For the PCR chamber fabrication, the circular chamber design was patterned on a Cr/Au deposited glass slide using the same procedure as described above, using photolithography and metal etching. To build an 80 µm deep chamber in the glass slide, the glass slide selectively covered with the Cr/Au layer (etch mask) was etched using hydrofluoric acid (49% HF, J.T. Baker, USA). Prior to this step, an adhesive tape (MicroAmp® Optical Adhesive Film, Applied Biosystems, Beverly, MA, USA) was attached to the back of the glass slide, allowing only the front side of the glass slide to be etched. The remaining metal etch mask layer was then removed by Cr and Au etchants and rinsed with deionized (DI) water. Since each glass slide (50.8×50.8mm^2^) contains four identical PCR chips (25.4×25.4mm^2^), a laser micromachining equipment (PLS 6.120D, Universal Laser Systems, Inc., Scottsdale, AZ, USA) was used to cut the single glass slides into four pieces. Before assembling the heater and the reaction chamber glass slides, inlet and outlet holes were drilled on the reaction chamber glass slide using a platinum coated drill bit (UKAM Industrial Superhard Tools, Valencia, CA, USA). To prevent possible cracking of the glass slides during drilling, a supporting glass slide was attached to the reaction chamber glass slide using wax (CrystalBond™, SPI, West Chester, PA, USA). After drilling the holes, the supporting glass slide was removed and the remaining wax on the reaction chamber glass slide was cleaned with acetone. Bonding between the heater glass slide and the PCR chamber glass slide was conducted using a UV curable adhesive (Loctite® 3492, Henkel, Rocky Hill, CT, USA). The final PCR microchip was then sterilized using an autoclave before use.

**Compact fluorescence detector.** The optical setup used in the real-time microchip PCR system is based on the detection of fluorescence dyes that intercalate with DNA, where fluorescent intensity is proportional to the amount of amplified DNA in the reaction chamber of the PCR microchip. The dye is excited with light emitted from an LED, and the emission light passing through series of filters and lenses to eliminate excitation light is detected through a photomultiplier tube (PMT) (Figure 1C). SYBR Green dye (QIAGEN, Valencia, CA, USA) was used as a fluorescence dye, which absorbs blue light (497 nm) and emits green light (520 nm). As a light source to excite the SYBR Green, a blue LED (470 nm, NSPB310B, Nichia, Tokushima, Japan) was used. The excitation filter (ET470/40x, Chroma Technologies, Brattleboro, VT, USA) was placed after the blue LED to further narrow down the spectrum, and a dichroic mirror (495DCLP, Chroma Technologies) was used to reflect the excitation light vertically to the PCR microchip placed on top of the optical housing. An aspheric lens (352330-A, Thorlabs, Inc., Newton, NJ, USA) was placed between the dichroic mirror and the PCR microchip to focus the excitation light onto the reaction chamber. The emission filter (ET535/50 m, Chroma Technologies) was placed before the PMT to transmit only the emitted light from the PCR sample. As a fluorescence detector, a compact PMT (H10721, 5×2×2 cm^3^, Hamamatsu, Hamamatsu City, Japan) was utilized because of its superior sensitivity compared to photodiodes even though the size and cost is higher. Since the amplicon detection is based on fluorescent intensity, drift in baseline readout of the PMT could result in inaccurate measurement of amplicon fluorescent intensity. Therefore, the PMT was warmed up for 1 hr by applying a working voltage of 5 V before any real-time PCR runs so that the degree of this drift becomes negligible. 

The housing for enclosing all of the above optical components was fabricated using a 3D material printer (ULTRA®, Envisiontec Inc., Dearborn, MI, USA). The overall size of the optical detector housing was 48×68×77 mm^3^. To characterize the performance of the fabricated compact fluorescence detector, eight different concentration of fluorescein isothiocyanate (FITC) dye from 0 nM (DI water) to 2000 nM were loaded into the reaction chambers of PCR microchips placed on top of the compact fluorescence detector housing and the fluorescent intensity was measured.

**Microcontroller for PCR thermocycle control and data acquisition.** In order to create a smaller and truly portable real-time PCR system, a compact battery-operated microcontroller unit (MCU) board was developed to control the thermocycling of the microchip as well as data acquisition and data display. The developed board is composed of an MCU and a custom-built printed circuit board (PCB) for the MCU. An 8-bit CMOS flash model microcontroller (PIC16F877, Microchip Technology Inc., Austin, TX, USA) was selected as the MCU and was programmed using the CCS-C language on the MPLAB IDE software suite (Microchip Technology Inc.) to regulate all operations of the system (thermocycling control, LED excitation light control, data acquisition from the PMT, and data display). Figure S1 shows the PCB schematic.

For thermocycling control, a proportional-integral-derivative (PID) control scheme was used, which measures the difference between current and target temperatures, and then change the current temperature to minimize this difference. To read the temperature of the reaction chamber, a fine-tip thermocouple (K-type, OMEGA, Stamford, CT, USA) was attached on the glass slide of the microchip (1 mm apart from the reaction chamber) using thermal grease (Thermalcote, Aavid Thermalloy, Concord, NH, USA) to form a tight thermal contact. Temperature reading from the thermocouple was converted and amplified to 5 mV/°C by an analog to digital (A/D) converter (AD8495, Analog Devices, Norwood, MA, USA), and this value was transmitted to an analog port of the MCU. This temperature information was then used to process the PID control for adjusting the pulse-width modulation (PWM) duty cycle of the current flow. By controlling a PSMNR5-40PS transistor (NXP semiconductor, Eindhoven, Netherlands), which was used as a switch, the MCU could control the current flow from a voltage source (15 V) to the heater. The PWM control signal from the MCU was sent to the gate of the transistor to switch the current flow. For faster cooling of the PCR microchip during thermocycling to minimize the total run time, a cooling fan (GB1206PHV1-AY, Digi-Key, Thief River Falls, MN, USA) was attached on top of the fluorescence detector housing as illustrated in Figure 1A and connected to the voltage source (15 V) and another PSMNR5-40PS transistor to let the MCU control turn on and off the cooling fan.

For fluorescent intensity measurement, the blue LED was turned on for 1 sec and then off by the MCU controlled 3.2 V voltage source during each thermocycle to minimize photobleaching. The emission light from the LED-excited amplicon was read by the PMT, PMT output current converted to a voltage value by an op-amp (OPA350, Texas Instruments, Dallas, TX, USA), and then acquired through the analog input port of the MCU.

For data display, a 128×64 pixel graphic LCD display screen (NHD-12864WG-BTGH-T#N, Newhaven Display, Elgin, IL, USA) controlled by the MCU was used. This display shows all necessary PCR process parameters such as the current temperature of the microchip, the current step and cycle number, the current PMT voltage reading of the present cycle’s fluorescence intensity, the increased amount of the current PMT voltage compared to the initial PMT voltage and a graph of the PMT voltage vs. cycle number in real time.

The overall real-time microchip PCR system requires 5 V for the microcontroller, A/D converter, PMT, op-amp, and LCD display, and 15 V for the heater and cooling fan. For the LED and the gain-control of the PMT, 3.2 V and 0.6 V are used, respectively. To make the system portable, a 2200 mAH 15 V Li-ion battery (Tenergy, Fremond, CA, USA) in conjunction with a 5 V voltage regulator (LM7805, Fairchild Semiconductor, San Jose, CA, USA) provides all voltages and power to the system. A voltage divider using two resistors was used to provide 3.2 V and 0.6 V from the 5V regulator. All circuit components are placed on a PCB designed using an electronic design automation (EDA) software tool (open source program, www.kicad-pcb.org) and fabricated by a PCB manufacturer (Advanced Circuits, Aurora, CO, USA). In parallel, during the initial device characterization process, a laptop-based version using NI modules (NI 9481 and NI 9219, National Instruments, Austin, TX, USA) was also built. An NI relay module (NI 9481) was used to control (on/off) the heater of the PCR microchip, the cooling fan, and the LED light. An NI data acquisition module (NI 9219) was used to monitor the microchip temperature and the PMT output voltage.

### Plant Pathogens and Sample Preparation

**Plant pathogen strains.** As a proof-of-concept, we selected *Fusarium* species and *Pseudomonas* species as fungal and bacterial pathogens, respectively, for this study (Table 1). Fungal strains stored in 30% glycerol at –80°C were activated on V8 juice agar (200 ml of V8 juice, 3 g of CaCO_3_, and 20 g of agar per liter) at 25°C for 7 days. Bacteria strains stored in 20% glycerol at –80°C were cultured on King’s B (KB) agar medium at 26°C for 2 days prior to experiments. Fungal strains were inoculated in 100 ml of yeast extract-peptone-dextrose (YEPD) broth with constant shaking (100 rpm) at 25°C under 14 h light/10 h dark photocycle. Fungal mycelia (50 mg) was harvested and used to extract DNA samples for PCR. For bacterial DNA samples, bacterial colonies were inoculated into 5 ml of nutrient broth-yeast extract (NBY) broth with shaking (180 rpm) at 26°C to an OD_600_ of 0.6 (∼5×10^8^ CFU/ml). Approximately 50 mg of bacterial cell mass was used for DNA extraction. In addition to *Pseudomonas* species, bacterial pathogen *Burkholderia glumae* was grown in liquid TNB (5 g trypton, 2.5 g yeast extract, 1 g dextrose, 8.5 g NaCl, and 4 g KNO_3_ per liter) culture until the bacterial cells reached late log phase (18 h, OD_600_ < 1.2).

**Table 1:** Pathogens tested with the real-time microchip PCR system.

Pathogen	Source
***Fusarium oxysporum*** ** spf. ** ***lycopersici***	
**Field isolates**	**Texas field isolates**
**ATCC 34298 ™**	**ATCC, Manassas, VI, USA**
***Fusarium verticillioides***	**FGSC, Kansas City, MO, USA**
***Pseudomonas syringae*** ** pv. ** ***syringae B728a***	**Quigley & Gross ** [**38**]
***Pseudomonas syringae*** ** pv. ** ***tabaci*** ** ATCC 11528**	**Quigley & Gross ** [**38**]
***Pseudomonas syringae*** ** pv. ** ***tomato*** ** DC3000**	**Quigley & Gross ** [**38**]
***Burkholderia glumae***	**Texas field isolate**

**DNA extraction.** Fungal and bacterial genomic DNA was isolated using the Plant/Fungi DNA Isolation Kit (Norgen Biotek Corp., Thorold, ON, Canada) following the manufacturer’s protocol with modifications to meet our needs for field application. A typical plant/microbe DNA extraction protocol calls for the use of liquid nitrogen and fine grinding of tissue materials. Since such steps are not suitable for field operation, our aim was to implement a modification that can provide a field-compatible but highly efficient DNA extraction method. *Fusarium* species and *Pseudomonas syringae* pv. *syringae* (*Pss*) B728a cells were macerated without liquid nitrogen in 500 μl of lysis buffer (1∶10 ratio, w/v), and treated with 100 (l of lysis additive buffer. The solution was vortexed and incubated at 65°C for 15 min, mixed with 100 (l of binding solution and incubated on ice for 10 min. Cell debris was precipitated by centrifugation for 10 min at 14,000 rpm, and the supernatant was mixed with an equal volume of 70% ethanol. The DNA-containing supernatant was added onto a column for binding, subsequently centrifuged, and washed twice with 500 μl of washing buffer. DNA was eluted by adding 50 μl of elution buffer, centrifuged for 2 min at 2,000 rpm, and for 1 min at 14,000 rpm. For maximum elution, another 50 µl of elution buffer was added to the column, and centrifuged for 2 min at 14,000 rpm. Other conventional extraction methods such as standard genomic DNA extraction method using liquid nitrogen and phenol/chloroform [28] and DNeasy® Plant Mini Kit (Qiagen, Valencia, CA, USA) were used to compare the DNA yield. *B. glumae* DNA extraction was performed in our laboratory following the established CTAB protocol [29], [30]. DNA quality and yield were determined on agarose gels and with a spectrophotometer.

### PCR Analysis Method

**Primer design and conventional Quantitative Real Time (qPCR) analysis.** *Fusarium oxysporum* f. sp lycopersici (Fol) isolates were subject to PCR analysis with primers designed to target a conserved locus SIX1 (Secreted In Xylem 1) [31]. All PCR primers used in this study are listed in Table 2. Here, we modified the primer set published by Van Der Does et al. [32] in order to generate a smaller amplicon for detection in our microchip system. Primers P12-F2B [32] and FOL-SIX1-R1 were used to amplify a 260-bp fragment from the SIX1 coding region. For *F. verticillioides* (Fv), β-tubulin gene (TUB2) was targeted for detection using β-tub-rt1 and β-tub-rt2 primer set [33]. Primer specificity and sensitivity were tested in a real-time PCR system (Cepheid Smart Cycler system, Sunnyvale, CA, USA). PCR was carried out in a 20 µl volume, containing 10 µl of 2× QuantiTect SYBR® Green PCR Master Mix (Qiagen), 20 pM of each primer and genomic DNA. Thermal cycling conditions consisted of 15 min at 95°C (hot-start step), followed by 35 cycles of 15 sec at 94°C (denaturation step), 30 sec at 60°C (annealing step) and 30 sec at 72°C (extension step).

**Table 2:** List of primers used in this study.

Primer	Sequence (5′→ 3′)	Target	Amplicon (bp)	Reference
**P12-F2B**	**TATCCCTCCGGATTTTGAGC**	***SIX1***	**260**	**Van Der Does ** ***et al.*** **** [**32**]
**FOL-SIX1-R1**	**TCGTTTCCAGGAAAGCTGC**	***SIX1***		**This work**
**β-tub-rt1**	**CAGCGTTCCTGAGTTGACCCAACAG**	***TUB2***	**200**	**Choi ** ***et al.*** **** [**33**]
**β-tub-rt2**	**CTGGACGTTGCGCATCTGATCCTCG**	***TUB2***		**Choi ** ***et al.*** **** [**33**]
**BF**	**CCGCGCTGTTCATGAGGGATAA**	***RHS***	**138**	**Kim ** ***et al.*** **** [**29**]
**BR**	**CGGGCGGAACGACGGTAAGT**	***RHS***		**Kim ** ***et al.*** **** [**29**]
**cmaAF**	**ACCGTGATGTTTACCTCTGGCA**	***cmaA***	**177**	**Winsor ** ***et al.*** [**35**]
**cmaAR**	**GCAGCGGTACCCAAACTTCAAA**	***cmaA***		**Winsor ** ***et al.*** [**35**]
**syrB2F**	**TCCTTATCGATCTGCAACTGGCGA**	***syrB2***	**160**	**Winsor ** ***et al.*** [**35**]
**syrB2R**	**AATGGTTGCCTGCAGTTCATTCCC**	***syrB2***		**Winsor ** ***et al.*** [**35**]
**tblAF**	**ACCCAGACTTAGAGCTCAAGCCAA**	***tblA***	**200**	**Winsor ** ***et al.*** [**35**]
**tblAR**	**TTCGATCTTGAAGCCAGCCTCAGT**	***tblA***		**Winsor ** ***et al.*** [**35**]

*Pss* B728a, *P. syringae* pv. *tabaci* (*P.s. tabaci*) ATCC 11528 and *P. syringae* pv. *tomato* (*Pst*) DC3000 were subjected to PCR with primers designed to target the conserved genes *syrB2* (syringomycin biosynthesis), *cmaA* (coronatine biosynthesis) and *tblA* (tabotoxin biosynthesis), respectively [34], [35]. *cmaA*, *syrB2*, and *tblA* amplicons were 177-, 160-, and 200-bp in size, respectively [35]. PCR was carried out in a 20-µl reaction mixture containing 10 µl of the SYBR® Green ER™ SuperMix Universal (Invitrogen, Grand Island, NY, USA), 8.16 µl nuclease-free water, 0.4 µl of each primer (200 nM), and 0.04 µl of ROX reference dye and genomic DNA. Thermal cycling conditions consisted of 20 sec at 95°C, followed by 40 cycles of 3 sec at 95°C, 30 sec at 60°C, and 30 sec at 72°C.

Real-time PCR of *B. glumae* was carried out with primers BF and BR [29] to amplify the *rhs* family gene (YD repeat protein) that produces a 138-bp amplicon. SYBR® Green PCR Master Mix (10 µl), 4 pmol of each primer, and 1 µl of extracted genomic DNA were combined for a total reaction mix of 20 µl. Real-time PCR was carried out with the following conditions: 20 sec at 95°C followed by 40 cycle of 95°C for 3 sec and 58°C for 30 sec, and a dissociation cycle of 95°C for 15 sec, 60°C for 1 min and 95°C for 15 sec.

**Figure 2:**
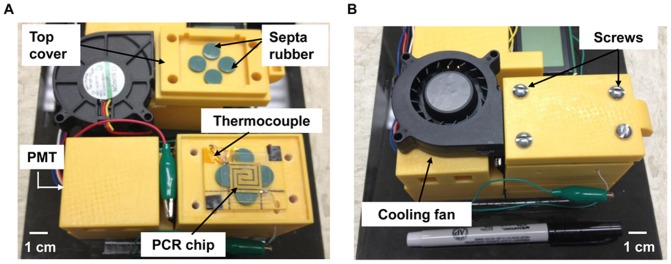
Photographs of the compact fluorescence detector housing assembly for real-time detection of amplified DNA samples. (A) A PCR chip with a thermocouple placed on top of the optical detector housing (bottom part of the image) and a cover integrated with a cooling fan and having septa rubbers to seal the inlet and outlet of the PCR chip (top part of the image). This cover also completely encloses the PCR chip to prevent ambient light from affecting the reading of the PMT. (B) The fully assembled housing that encloses the PCR microchip. The cooling fan can be seen on top of the housing, as well as screws that provide the tight seal.

**Real-time microchip PCR.** All samples for the PCR mixture (SYBR® green, primers, and DNA sample) were stored at –20°C and thawed on ice before use. A 20-µl PCR sample was prepared by mixing 13.5 µl of a QuantiTect SYBR® Green PCR mix, 1 µl of forward and reverse primers, 1 µl of 10% polyvinylpyrrolidone (PVP), 1 µl of 1 mg/ml bovine serum albumin (BSA) and 2.5 µl of DNA sample. Sample (8 µl in volume) was loaded into the reaction chamber of the PCR microchip using a pipette and the microchip was placed on top of the fluorescence detector housing (Figure 2A). Both the inlet and outlet of the PCR microchip were sealed with green septa rubbers (Thermogreen LB-2, Sigma-Aldrich, St. Louis, MO, USA) to prevent evaporation during PCR. A top cover with a cooling fan was then placed on top of the microchip (Figure 2A) and tightened by screws in order to tightly seal the microchip (Figure 2B). Thus, the entire microchip is enclosed inside a plastic casing, providing a dark environment to minimize background optical noise during fluorescence detection.

Thermal cycling condition was 15 min at 95°C (hot-start step), followed by 35 cycles of 50 sec at 94°C (denaturation step), 60 sec at 60°C (annealing step) and 60 sec at 72°C (extension step). The emission signal from the PCR microchip was monitored in real time through the LCD display on the PCB. To verify the PCR results, amplified samples were extracted from the microchip and were subjected to electrophoresis in a 1.2% agarose gel.

## Results and Discussion

### Simulation & Fabrication Results

**Figure 3:**
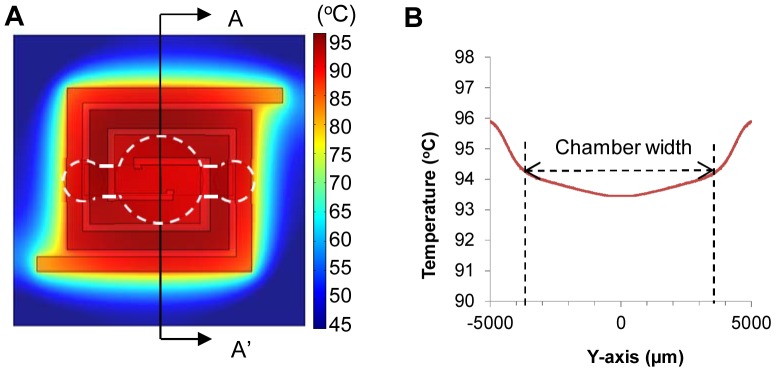
Simulated temperature profile of the microchip. (A) COMSOL Multiphysics® simulation showing uniform temperature distribution in the PCR chamber region when heated to 94°C (White dotted line shows the position of the reaction chamber). (B) Temperature profile across A-A’ shows the uniformity of the temperature in the chamber region of the PCR microchip within 1°C variation when the PCR microchip is heated to 94°C.

Thermocycle characterization of the PCR microchip. The temperature distribution over the entire PCR heater, obtained from COMSOL Multiphysics® simulation, showed very good uniformity (Figure 3A). Detailed temperature profile inside the chamber area was further analyzed (Figure 3B), which shows uniform temperature within 1°C variation, demonstrating the capability of the heater. When a voltage of 15 V was applied, the PCR sample inside the microchip reached 95°C from room temperature within 11 sec. The time needed for the annealing temperature (60°C) to reach the extension temperature (72°C) was 3 sec, and time needed from the extension temperature to the denaturation temperature (94°C) was 4 sec. The cooling time from the extension temperature to the annealing temperature was 60 sec without a cooling fan and 14 sec when using a cooling fan. Since the cooling-time difference was significant (46 sec) and corresponds to 27 min difference in the overall assay time over 35 thermocycles, we decided to use a cooling fan in our real-time microchip PCR system. The total PCR time for 35 cycles (a single cycle: 50 sec at denaturation, 60 sec at annealing, and 60 sec at extension), including a 15 min initial hot-start step at 95°C, was 2 hr. 78% (99 min) of the total PCR time was taken for steps in 35 cycles, 12% (15 min) for the initial hot-start step, and 10% (12 min) for heating and cooling between each step. The total PCR time can be reduced by shortening the heating and cooling time between each step by using a smaller reaction chamber, however the reduction in time would only be 6 min if the heating and cooling time is reduced by 50%.

**Compact fluorescence detector.** When using nine different concentrations of FITC dye from 0 nM to 2000 nM, the PMT reading shows linear output (R^2^ = 0.9991) from 600 mV to 925 mV (Figure S2). Thus, the compact fluorescence detector shows sufficient sensitivity for low limit of detection as well as sufficient linearity for quantification.

**Figure 4:**
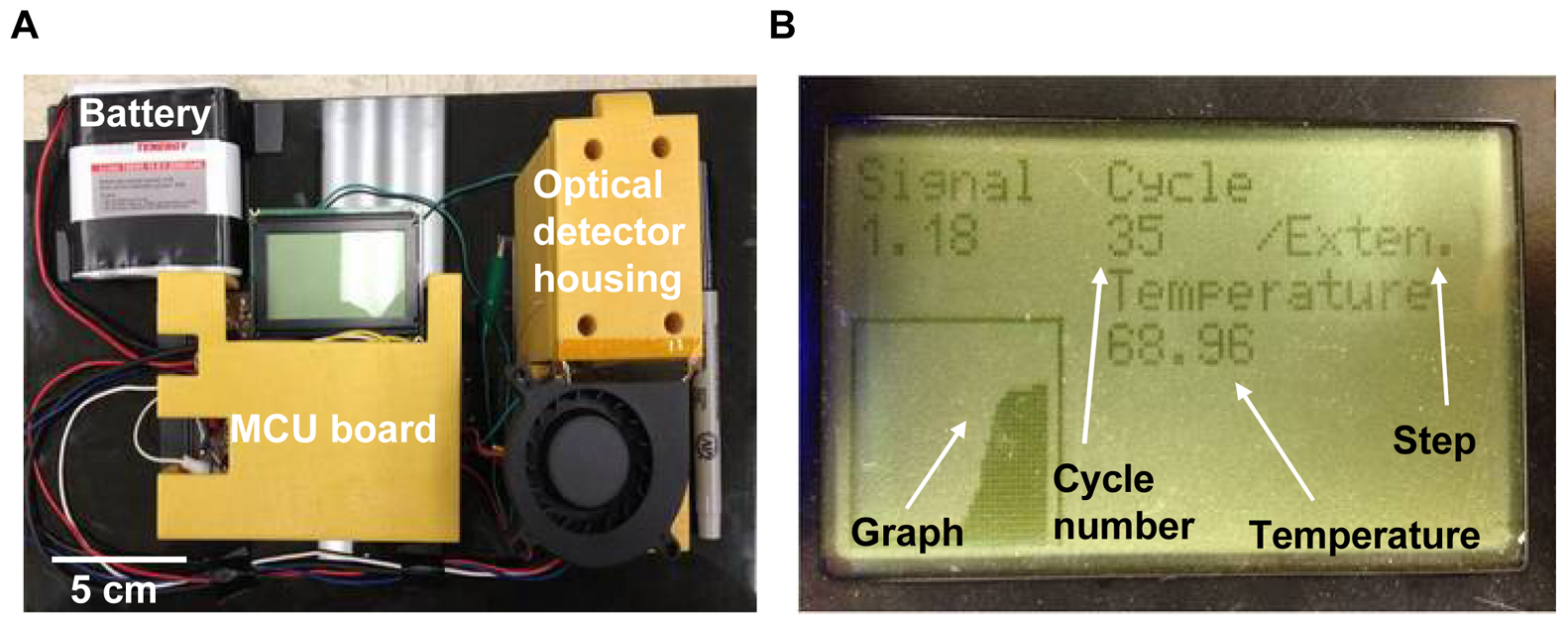
Photograph of the portable real-time microchip PCR system. (A) The portable real-time microchip PCR system controlled by an MCU and powered by a battery. The entire size is 16×28×9 cm^3^, and the total weight is 843 g. (B) The LCD of the MCU board displays several information about the real time PCR such as the number of cycle, current PCR step, current temperature, fluorescence intensity, the increasing amount of the present cycle’s fluorescence intensity compared to the first cycle’s fluorescence intensity, and the graph showing the trace of the fluorescence intensity of each cycle.

**Overall system assembly.** Figure 4A shows the real-time microchip PCR system with a compact fluorescence detector, fully controlled by an MCU module and powered by a battery. The complete system fits in a 25×16×8 cm^3^ box and weighs less than 843 g. Critical processing information and results, including fluorescent readout from the PMT, current thermocycle number, current thermocycle step, and temperature of the reaction chamber were displayed on the LCD display (Figure 4B). The MCU module consumed about 110 mAh for one PCR run that includes one hot-start cycle and 35 PCR thermocycles, meaning that more than 20 PCR runs can be conducted from a single battery charge. The compact system size and efficient energy consumption of our system makes this system more suitable for on-field plant pathogen diagnostics than other portable real-time PCR systems that still require additional instrumentations such as a laptop or additional control modules [14]–[15].

### DNA Preparation and PCR Performance

**Development of a DNA extraction method feasible for field application.** We modified a variety of publicly and commercially available DNA extraction methodologies and kits to be usable in the absence of liquid nitrogen. Our test results show that a commercial Plant/Fungi DNA Isolation Kit when used with our unique modifications explained in the materials and methods (section 2.2) provided high quality and sufficient yield of genomic DNA from fungi and bacteria. With these modifications, we were able to improve the DNA yield close to the levels with either liquid nitrogen or chemical usage, obtaining 13 µg and 65 µg of DNA per 50 mg of fungal and bacterial biomass, respectively (Table 3). While the quantity of microbial genomic DNA extracted by the improved method is not as effective as conventional in-lab protocols, the yield obtained was sufficient for PCR based diagnostics.

**Table 3:** Comparison of fungal and bacterial genomic DNA extracted by a variety of methods with or without the use of liquid nitrogen, phenol and chloroform.

Organism a	Kit	Liquid nitrogen	Phenol/ Chloroform	Modifications b	Yield (µg) c
***Fol***	**Standard method**	**Yes**	**Yes**	**None**	**33±2.03**
	**Standard method**	**No**	**Yes**	**Yes**	**8.5±0.8**
	**Norgen**	**No**	**No**	**None**	**6.7 ±0.8**
	**Norgen modification (our method)**	**No**	**No**	**Yes**	**12.6±2.02**
***Pss***	**Standard method**	**No**	**Yes**	**None**	**52.4±1.18**
	**DNeasy**	**No**	**No**	**None**	**2.2±0.6**
	**Norgen modification (our method)**	**No**	**No**	**Yes**	**64.76±7.0**

*Fusarium oxysporum* f. sp. *lycopersici* (FOL) and *Pseudomona syringae* pv. *syringae* (*Pss*) genomic DNA was isolated from 50 mg of wet fungal and bacterial biomass, respectively.^a^

b Modifications as described in the materials and methods (section 2.2).

c Values are the means of three biological replicates ± SE.

**Figure 5:**
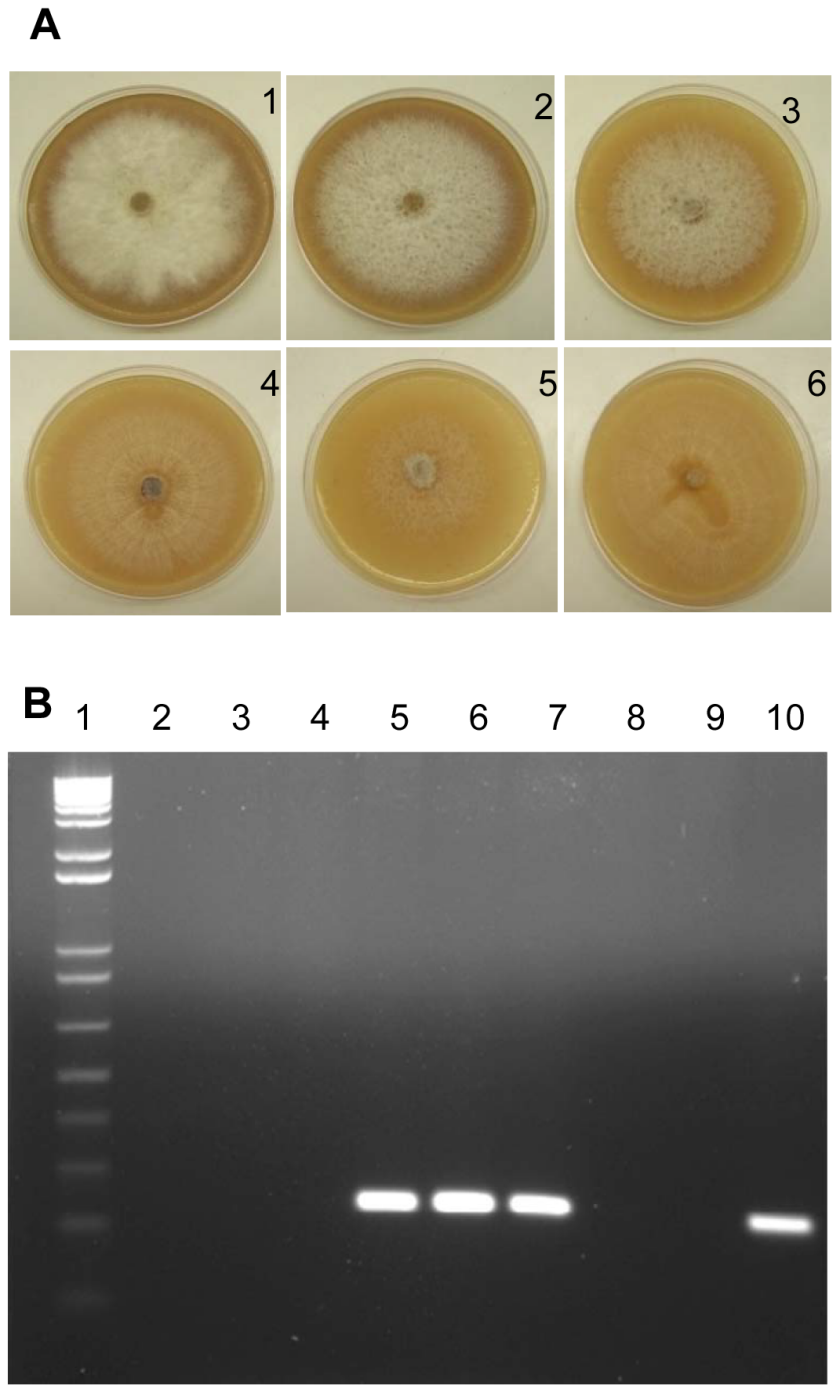
Primer Specificity of *SIX1* for *Fusarium oxysporum* f. sp. *lycopersici* (*Fol*) strains. (A) *Fusarium* strains were point inoculated with an agar plug (0.5 cm in diameter) on V8 agar medium and incubated for 8 days at 25°C under 14 h light/10 h dark cycle. 1–4: *Fol* strains; 5: *F. oxysporum* f. sp. *vasinfectum*; 6: *F. oxysporum*. (B) PCR analyses for *Fol* primers specificity. Lanes: 1, DNA marker; 2, water; 3, *F. verticillioides*; 4, *F. graminearum*; 5,6,7,10, *Fol* isolates; 8, *F. oxysporum* f. sp. *vasinfectum*; 9, *F. oxysporum.*

**Primer specificity and sensitivity.** *Fol* is a soil-borne fungus that causes tomato wilt worldwide. Three physiological races of *Fol* have been reported worldwide, where race 1 is most common in countries with warm climates. However, molecular diagnosis of these races is challenging since only the genome sequence of *Fol* race 2 is publicly available (http://www.broadinstitute.org/). *Fol SIX1* gene encodes for a virulence factor towards tomato plants, which is secreted in the xylem of the host during colonization [32]. Significantly, Six1 has been shown to be a key protein required for full virulence of the pathogen [36] and is a suitable target for diagnostics. Figure 5 shows that PCR analyses targeting a small fragment of *SIX1*, using primer sets P12-F2B and FOL-SIX1-R1 resulted in a clear and specific amplicon for *Fol* strains. In addition, no false-positive amplicons were observed when tested with other *Fusarium* species (Figure 5), as has been previously reported by Lievens *et al.* [31]. Using the DNA extraction method described earlier and primer sets P12-F2B and FOL-SIX1-R1, we determined that the limit of detection is 10 pg of gDNA/ real-time PCR.

There are over 50 pathovars of *P. syringae* that vary in host range and symptomology [34], [37]. For accurate diagnosis of disease caused by *P. syringae*, primers that can detect these pathovars with a high rate of specificity are critical. The gene *syrB2,* selected for the detection of *Pss* B728a, is known to be conserved among most *P. syringae* strains that produce syringomycin, syringotoxin, or syringostatin [34]. Table S1 shows the specificity of *syrB2* primers for the detection of *Pss* strains utilizing PCR and real-time PCR. The *syrB2* primers were also screened against *P. syringae* strains that were not producers of syringomycin, syringotoxin or syringostation. These strains were used as negative controls that produced no amplicons on an agarose gel (data not shown).

These results demonstrate that the primers designed for *Fol* and *Pss* are suitable for a reliable and sensitive identification method. Furthermore, due to the small size of amplicons used for conventional real-time PCR analysis (section 3.1 in the materials and methods), overall PCR running time can be decreased, resulting in faster detection of the pathogens.

**Performance of the portable real-time microchip PCR system.** To demonstrate the performance of our developed real-time microchip PCR system, we investigated the success rate and the detection limit of the system with *Fol* and *Fv* genomic DNA samples.

When loading sample, bubble formation occasionally occurred, but at a very low rate of about one per 20–30 loadings due to capillary force as well as the hydrophilicity of the chamber. When bubble formation was recognized, the PCR mixture was pulled and pushed from the reaction chamber using the same pipette with its tip placed in the inlet hole. In general, 2–4 repeated push/pulling motion using the pipette successfully removed the bubble.

Firstly, a negative control sample (no DNA) was amplified through 35 thermocycles, and the PMT showed voltage increase of 0.02±0.02 V between the 1^st^ thermocycle and 35^th^ thermocycle. The experiment was repeated five times. The voltage sum of the average and the standard deviation (0.04 V) from this negative control was considered as a threshold line to determine the presence of amplified DNA for all future tests. Subsequently, ten PCR tests with FOL genomic DNA (300 ng/sample) were conducted. Increases in PMT output voltage (0.13±0.2 V) of more than the threshold voltage were obtained from all ten tests, indicating that the portable real-time microchip PCR system successfully amplified and detected the target DNA consistently. The *Fv* genomic DNA (300 ng/sample) was tested seven times with 100% success rate, showing average increase in PMT output of 0.32±0.13 V. The *Pss* B728a DNA (75 ng/sample) was tested ten times and also showed 100% success rate through the PMT output increase (0.1±0.08 V). After these test results, we continued with *P.s. tabaci* ATCC 11528 (115 ng/sample), *Pst* DC3000 (166 ng/sample), and *B. glumae* (300 ng/sample and 150 ng/sample), each with four repetitions. All tests showed 100% success rate (Table 4).

**Table 4:** Analysis of pathogen DNAs using the real-time microchip PCR system.

Sample	Cq	Success rate
***Fol*** ** 300 ng/sample**	**18**	**100% (** ***n*** ** = 10)**
***Pss*** ** B728a 75 ng/sample**	**23**	**100% (** ***n*** ** = 10)**
***P.s. tabaci*** ** ATCC11528 115 ng/sample**	**23**	**100% (** ***n*** ** = 4)**
***P.s.t*** **. DC3000 166 ng/sample**	**17**	**100% (** ***n*** ** = 4)**
***B. glumae*** ** 300 ng/sample**	**18**	**100% (** ***n*** ** = 4)**
***B. glumae*** ** 150 ng/sample**	**21**	**100% (** ***n*** ** = 4)**
**Negative**	**-**	**- (** ***n*** ** = 5)**

**Figure 6:**
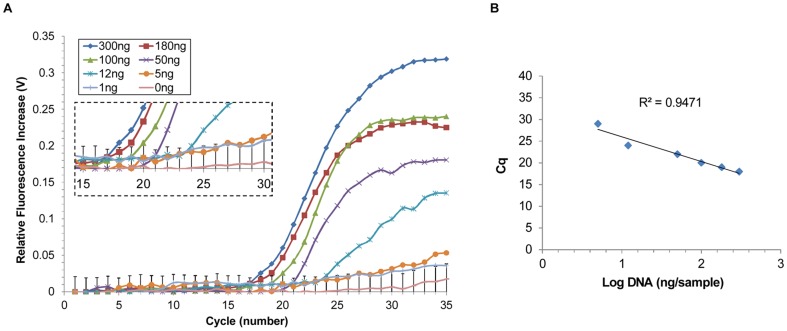
Performance of the portable real-time microchip PCR system using different concentrations of *Fv* DNA. (A) Amplication plot of PMT voltage output over the thermocycle number. Using 300, 180, 100, 50, 12, and 5 ng/sample, the Cq was 18, 19, 20, 22, 24, and 29, respectively (enlarged graph shows the Cq). The error bar of the negative control (0 ng) is the standard deviation. (B) Correlation graph of the DNA amount and the Cq (R^2^>0.947).

To determine the detection limit of our system, seven different concentrations of *Fv* DNA – 300, 180, 100, 50, 12, 5, and 1 ng/sample – were tested with four repetitions. The PMT voltage increase versus the number of thermocycles was plotted (Figure 6a) and cycle of quantification (Cq) values were extracted from the graph. The Cq value was defined as the thermocycle number when the PMT output increased over the previously determined threshold line of 0.04 V. PMT outputs from the concentrations of 300, 180, 100, 50, 12, and 5 ng/sample all became larger than the threshold, while the PMT output from the 1 ng/sample was not over the threshold. Thus, the detection limit of our developed real-time microchip PCR system was determined to be 5 ng/sample. We expect that this detection limit can be further improved in the future by using a higher power excitation light source, more efficient filter sets (transmission efficiency larger than the currently used 80%), and a more sensitive PMT with lower background noise (luminous sensitivity larger than the currently used 350 µA/lm), all currently commercially available.

The Cq values of *Fv* DNA 300, 180, 100, 50, 12, and 5 ng/sample were 18, 19, 20, 22, 24, and 29, respectively, which were correlated to the amount of DNA (Figure 6b). As the amount of DNA in the sample solution decreased, corresponding Cq values increased, which indicate that more thermocycles are required for the target DNA to be sufficiently amplified to have fluorescent signals higher than those from the negative control (Table 4).

**Figure 7:**
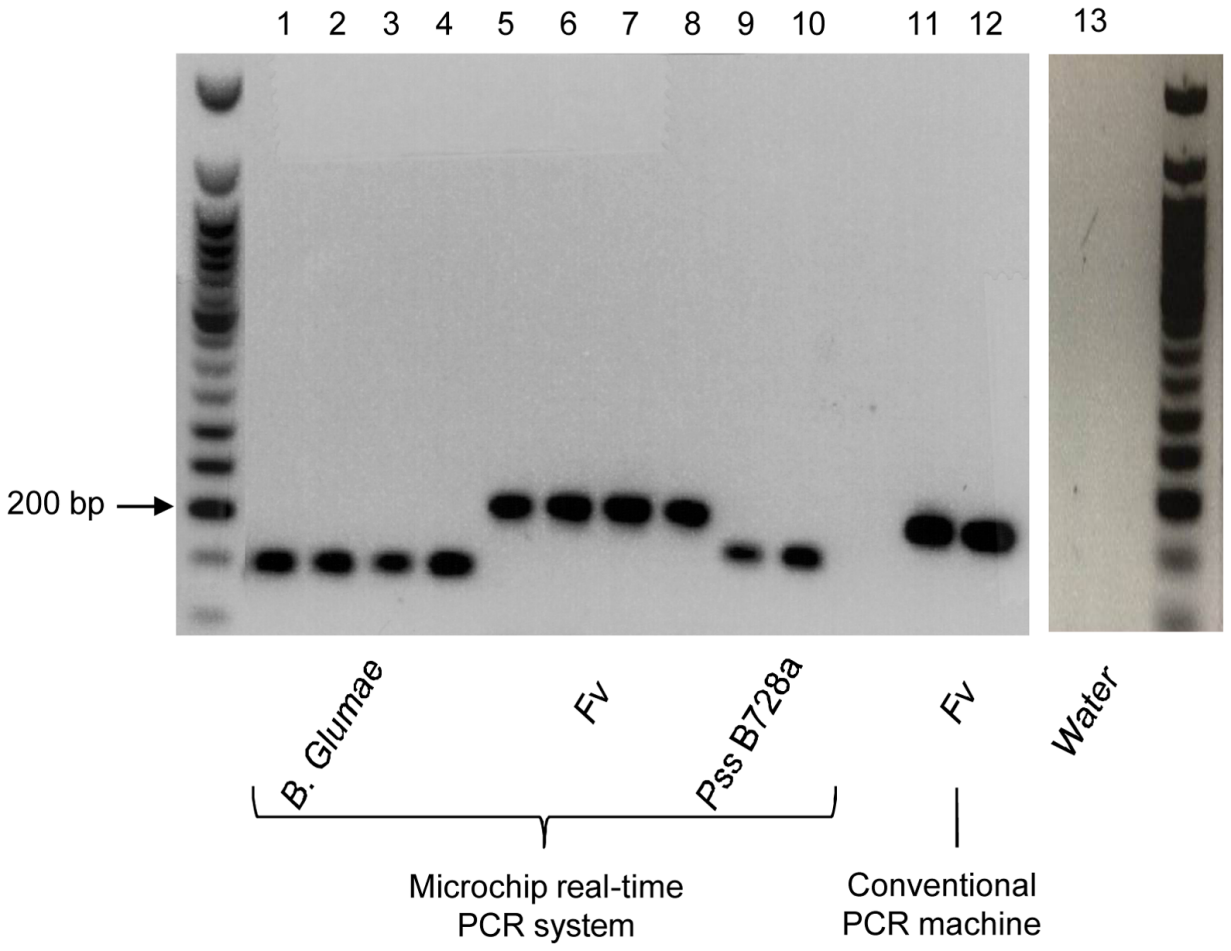
Gel electrophoresis result to verify the portable real-time microchip PCR system. The picture shows strong bands of *B. glumae* DNA (150 ng/sample), *Fv* DNA (50 ng/sample), and *Pss* B728a DNA (75 ng/sample) amplified using the real-time PCR microchip system (sample 1 to 10). *Fv* DNA 50 ng/samples amplified using a conventional PCR machine and water were used as control (sample 11 to 13).

Throughout our experiments, resulting amplicons from the real-time PCR microchips were extracted and subjected to conventional gel electrophoresis. All successful real-time microchip PCR runs as determined by PMT output voltage increase also showed strong gel bands, validating our real-time microchip PCT result. Figure 7 shows examples of such confirmation results, including real-time microchip PCR results from *Fv* DNA (50 ng/sample), *Pss* B728a DNA (75 ng/sample), and *B. glumae* DNA (150 ng/sample). This confirmation also allows us to reduce the PCR reaction chamber volume of the microchip in the future, since the current chamber volume (8 µl) was simply determined based on the needed sample amount for off-chip gel electrophoresis conformation.

Since we have initially used a much longer step time (50 sec at 95°C, 60 sec at 60°C, 60 sec at 72°C) to amplify our particular plant pathogen samples compared to those of other microchip PCR systems (typically using 15 sec at 95°C, 30 sec at 60°C, 30 sec at 72°C), we attempted to further optimize and reduce this PCR step time to reduce the overall assay time. Successful amplification of *Fv* DNA was obtained by using as short as 20 sec at 94°C, 30 sec at 60°C, and 30 sec at 72°C (Figure S3). This resulted in the reduction in the total operation time to 1.2 hours, including the initial hot-start step (15 min), a 0.8 hour reduction, thus making it much more suitable for field applications.

## Conclusion

We developed a portable and standalone real-time microchip PCR system equipped with a microcontroller for controlling the entire PCR operation and with a compact fluorescence detector for acquiring fluorescence emission intensity for on-field plant disease diagnostics. This battery-powered system was fully operational with no need for an external computer or auxiliary power source. With these specifications, we were able to develop a real-time microchip PCR system that is 25×16×8 cm^3^ in size and 843 g in weight, substantially increasing the prospect of a fully functional yet truly portable real-time microchip PCR system. The MCU runs 35 thermocycles and detects amplicons in real time with low power consumption (110 mAh), enabling more than 22 runs with a single battery charge. We also developed efficient DNA extraction protocols for field operations, notably without the use of liquid nitrogen and other large lab equipment. Multiple fungal and bacterial genomic DNA samples were tested with our portable real-time microchip PCR system, and successful DNA amplification resulted in increased fluorescent signal through the PMT. We were extremely encouraged by the consistency of success in DNA amplification and detection (100% success rate and validation through gel electrophoresis). The detection limit of our system was 5 ng/sample DNA, with room for further improvement. Significantly, our portable lab-on-a-chip real-time PCR system, without the need for external computer, power supply or other apparatuses, has the potential to transform on-field plant disease diagnostics that can safeguard our food production and safety.

## Supporting Information

Figure S1 **Schematic of the compact battery-operated microcontroller (MCU) printed circuit board.** The MCU board controls the thermocycling of the microchip as well as the data acquisition and data display in order to create a smaller and truly portable real-time PCR system. The MCU board is composed of a 5 V regulator, an A/D converter, a current-to-voltage op-amp, transistor switches, and a LCD display.
(TIF)

Figure S2 **Characterization of the compact fluorescence detector.** FITC 0, 250, 500, 750, 1000, 1250, 1500, 1750, and 2000 nM were used. The PMT output shows linear increase from 600 mV to 925 mV.
(TIF)

Figure S3 **Performance of the optimized portable real-time microchip PCR system.** DNA amplification shown as PMT output voltage over the thermocycle number. Three different PCR step times were tested with *Fv* DNA (100 ng/sample). (1) 30 sec at 94°C, 40 sec at 60°C, and 40 sec at 72°C; (2) 20 sec at 94°C, 40 sec at 60°C, and 40 sec at 72°C; (3) 20 sec at 94°C, 30 sec at 60°C, and 30 sec at 72°C. The three numbers in the legend box are the time for denaturation, annealing and extension steps, respectively. The real-time PCR run was successful in all three experiments.
(TIF)

Table S1 **Primer specificity of** ***syrB2*** **for** ***Pseudomonas syringae*** **pv.** ***syringae*** **strains.**
(DOCX)
