# Supplementary figures and images for: Development of a Real-Time Microchip PCR System for Portable Plant Disease Diagnosis
Source: PLoS One. 2013 Dec 12;8(12):e82704. doi: 10.1371/journal.pone.0082704 (PMC3861469; doi:10.1371/journal.pone.0082704)

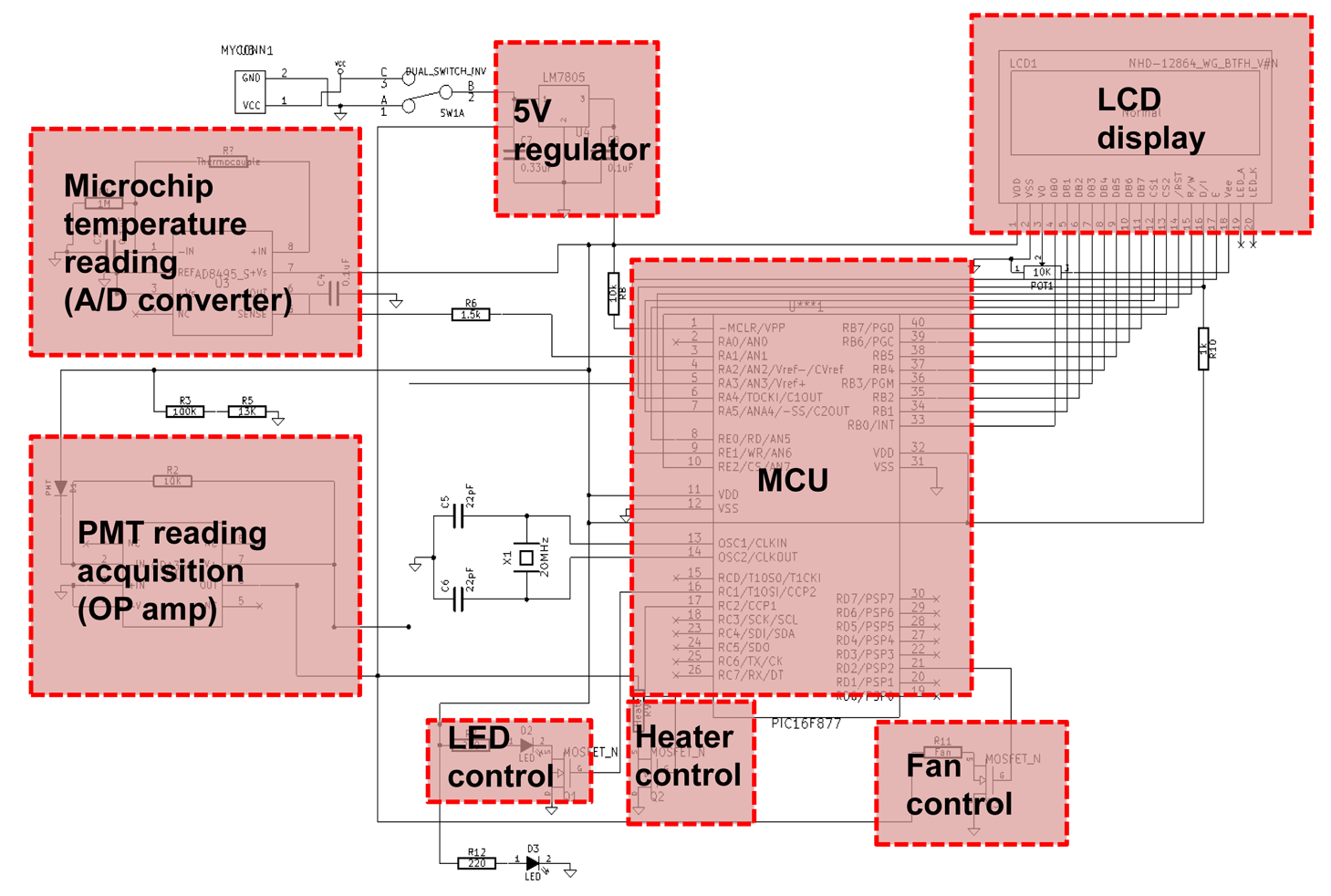

Supplement: Figure S1 — Schematic of the compact battery-operated microcontroller (MCU) printed circuit board. The MCU board controls the thermocycling of the microchip as well as the data acquisition and data display in order to create a smaller and truly portable real-time PCR system. The MCU board is composed of a 5 V regulator, an A/D converter, a current-to-voltage op-amp, transistor switches, and a LCD display. (TIF) [file pone.0082704.s001.tif]

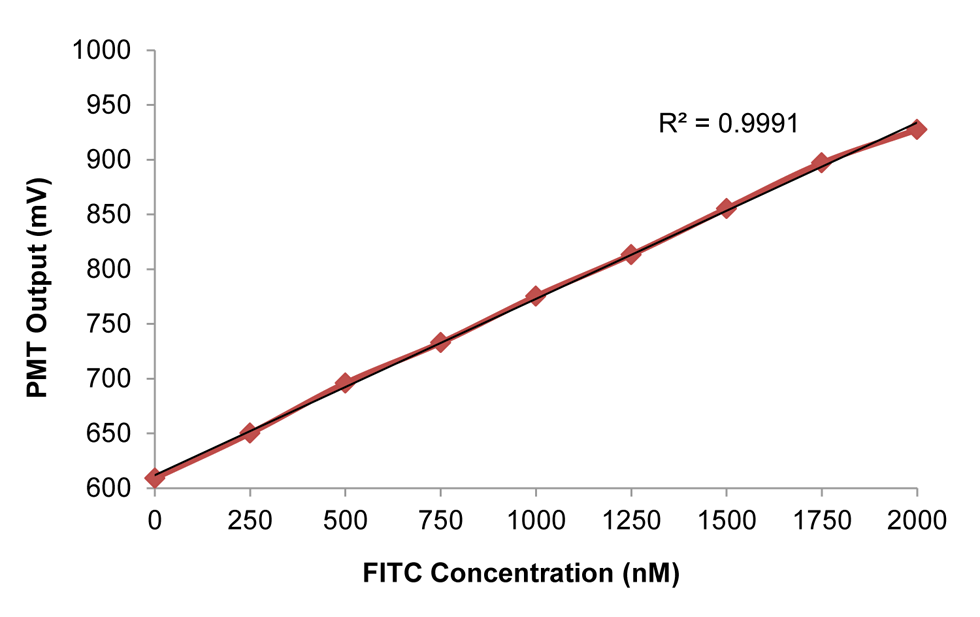

Supplement: Figure S2 — Characterization of the compact fluorescence detector. FITC 0, 250, 500, 750, 1000, 1250, 1500, 1750, and 2000 nM were used. The PMT output shows linear increase from 600 mV to 925 mV. (TIF) [file pone.0082704.s002.tif]

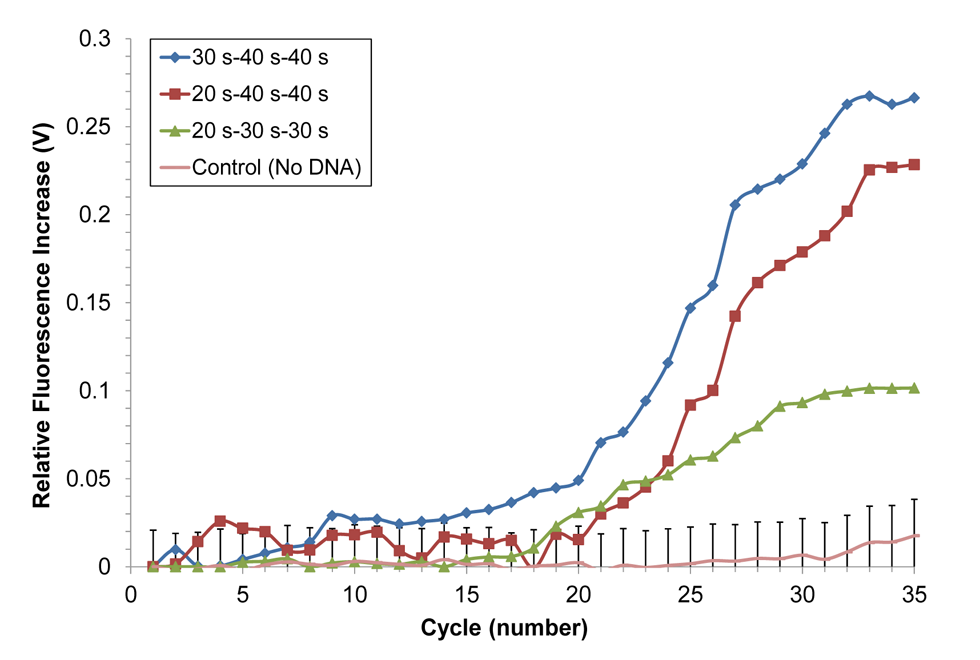

Supplement: Figure S3 — Performance of the optimized portable real-time microchip PCR system. DNA amplification shown as PMT output voltage over the thermocycle number. Three different PCR step times were tested with Fv DNA (100 ng/sample). (1) 30 sec at 94°C, 40 sec at 60°C, and 40 sec at 72°C; (2) 20 sec at 94°C, 40 sec at 60°C, and 40 sec at 72°C; (3) 20 sec at 94°C, 30 sec at 60°C, and 30 sec at 72°C. The three numbers in the legend box are the time for denaturation, annealing and extension steps, respectively. The real-time PCR run was successful in all three experiments. (TIF) [file pone.0082704.s003.tif]
