# Supplementary material for: Development of a Real-Time Microchip PCR System for Portable Plant Disease Diagnosis
Source: PLoS One. 2013 Dec 12;8(12):e82704. doi: 10.1371/journal.pone.0082704 (PMC3861469; doi:10.1371/journal.pone.0082704)
Supplement: Table S1 — Primer specificity of syrB2 for Pseudomonas syringae pv. syringae strains. (DOCX) [file pone.0082704.s004.docx]

**Supplementary Table S1.** Primer specificity of *syrB2* for *Pseudomonas syringae* pv. *syringae* strains.

| Strain | PCR | qPCR |
| --- | --- | --- |
| B728a  Pss 61  Pss268  445  761-5  B3A  B15+  B301D  B457  HS191  I4S1  Ps3  Ps17  Ps281  Ps12274  W4N03  W4N27  W4N42  W4N47  W4N50 | +  +  +  +  +  +  +  +  +  +  +  +  +  +  +  +  +  -  -  - | +  +  +  +  +  +  +  +  +  +  +  +  +  +  +  +  +  +  _  _ |
| W5N56  W4N86  W4N87  SD7  SD8  SD475 | -  +  +  +  +  + | +  -  -  +  +  + |
|  |  |  |
